# Participant experiences from chronic administration of a multivitamin versus placebo on subjective health and wellbeing: *a double-blind qualitative analysis of a randomised controlled trial*

**DOI:** 10.1186/1475-2891-11-110

**Published:** 2012-12-14

**Authors:** Jerome Sarris, Katherine H M Cox, David A Camfield, Andrew Scholey, Con Stough, Erin Fogg, Marni Kras, David J White, Avni Sali, Andrew Pipingas

**Affiliations:** 1 Department of Psychiatry, The University of Melbourne, Melbourne, Australia; 2 Centre for Human Psychopharmacology, Swinburne University of Technology, Hawthorn, Australia; 3 Brain and Psychological Sciences Research Centre, Swinburne University of Technology, Hawthorn, Australia; 4 National Institute of Integrative Medicine, Hawthorn, Australia

**Keywords:** Multivitamin, Mood, Energy, Cognition, Adverse reactions, Qualitative

## Abstract

**Background:**

While many randomised controlled trials have been conducted on multivitamins, to our knowledge no qualitative research exploring the subjective experience of taking a multivitamin during a clinical trial has been reported.

**Methods:**

Semi-structured and open-ended written questions were incorporated into a 16-week double-blind, randomised, placebo-controlled, parallel groups trial of once-daily multivitamin administration. At the final study visit (week 16), three open-ended questions were posed to elucidate any positive, negative or unusual experiences from taking either the multivitamin or matched placebo. Qualitative thematic analysis was undertaken by researchers who were blind as to treatment condition of participants, and triangulation (independent analysis from three researchers) was employed to ensure methodological rigour. Participant’s experiences were categorised as “positive” or “negative” and a Chi Square analysis was then applied to each of the experiential themes, to compare experiences between the multivitamin and placebo groups, (subdividing the groups by gender). Usual experiences were categorised and discussed separately.

**Results:**

Of the 182 participants enrolled, 116 completed the study and qualitative data were available from 114 participants. Thematic analysis revealed significant effects in favour of the multivitamin over placebo for participants experiencing increased energy levels (*p*=.022) and enhanced mood (*p*=.027). The beneficial effect on energy levels was particularly evident among female participants. A trend was found for participants reporting better sleep in the multivitamin over placebo. The multivitamin and placebo groups did not significantly differ in perceived positive or negative effects in areas relating to other aspects of mental function or physical health. No significant negative effects were revealed, although there was a non-significant trend for more people in the multivitamin group having minor digestive complaints.

**Conclusion:**

This represents the first documented qualitative investigation of participants’ experience of chronic administration of a multivitamin. Results uncovered a range of subjective beneficial effects that are consistent with quantitative data from previously published randomised controlled trials examining the effects of multivitamins and B vitamin complexes on mood and well-being.

**Trial registration:**

**Prior to commencement** this trial was registered with the Australian New Zealand Clinical Trials Registry ( http://www.anzctr.org.au) ACTRN12611000092998

## Background

The use of multivitamin (MV) supplements has become increasingly popular among the general public [1]. There is growing literature to suggest that MV or multinutrient supplementation may have cognitive and/or mood benefits in children [2] and across the adult life span [3-10]. There are a numerous ingredients commonly found in MV and mineral supplements that may affect cognition and mood. Low B vitamin levels have been linked to increased levels of homocysteine, which can have a detrimental effect on cognition [11,12], and lowered levels of Vitamin B12 and folic acid have been associated with higher incidence of depression or depressed mood [13-15]. Vitamin C may improve cognition, as a result of central antioxidant activity [16,17]. Vitamin D can improve mood [18], and supplementation may promote general health through its integral involvement in a vast number of biological processes [19]. Minerals such as zinc, magnesium and calcium influence neurotransmitter systems and therefore play an important role in maintaining healthy cognitive function and mood [20-22]. Additionally, though often present in small (and potentially sub-therapeutic) quantities in MVs, herbal ingredients such as *Ginkgo biloba* and *Panax ginseng* may reduce stress and anxiety [23,24] while *Centella asiatica* has exhibited mood enhancing effects [25].

The Swisse Ultivite F1 multivitamin® (SMV) contains a proprietary blend of vitamins at levels exceeding recommended daily intakes (which are arguably low), minerals, as well as low doses of a range of medicinal herbs. Previous studies from our laboratory have identified beneficial effects of SMV use in older populations. Harris et al. [7] found that an 8-week supplementation with Men’s SMV in males aged 50–69 years was associated with a significant reduction in symptoms of depression and anxiety and an improvement in alertness and general health, that were not seen in the placebo group. The same treatment was associated with improvements in contextual recognition memory performance in men aged between 50 and 74 years who had a sedentary lifestyle [10]. Macpherson et al. [26], also showed that 16-week supplementation with a SMV formula designed for older women (50+ Ultivite®) was associated with faster speed of spatial working memory performance.

The present 16-week double-blind randomised controlled trial (RCT) sought to extend these findings to younger individuals aged 20–50 years and to test once daily Swisse Men’s *and* Women’s Ultivite® multi-nutrient supplements. The clinical trial assessed the effect of the SMV on cognition, and on psychological attributes such as mood, stress, sleep, and energy levels. The study also utilised a “mixed-methods” approach employing both quantitative and qualitative methodological techniques (the quantitative analyses are reported elsewhere). Qualitative measures may form the focus of a study, or be part of a combined approach whereby a qualitative component augments the quantitative mainstay of an RCT [27-29]. These measures can assist to understand participants’ experiences of an intervention in an RCT [27-29]. This approach can help to develop hypotheses for future research, and may identify previously unknown therapeutic benefits or side-effects. To our knowledge qualitative methodology has not previously been used in RCTs of MVs. It is important to note that while there is a key difference between reductive statistically-based quantitative research and exploratory experiential qualitative research, in order to maintain methodological rigour, it is possible to conduct qualitative research in a double-blinded manner, with neither the participants nor the researchers who analyse the data knowing which participants received the active or placebo intervention.

In this paper we present the qualitative component of the study (analysed via quantitative methodology) which explores participants personal experiences of being chronically administered a MV compared to placebo.

## Methods

### Overview

Eligible consenting adults participated in a 16-week double-blind RCT involving once daily administration of SMV tablet or matching placebo. Participants attended three testing sessions at the Centre for Human Psychopharmacology at Swinburne University in Melbourne, at baseline, 8-weeks and 16-weeks, for the assessment of wellbeing, mood, stress and cognition. Potential biological mechanisms of action were also examined. The study was granted ethical approval by the Swinburne University of Technology Ethics Human Ethics Committee (SUHREC Project 2010/261) and was registered with the Australian New Zealand Clinical Trials Registry (ANZCTR no: ACTRN12611000092998) prior to commencement.

### Participants

Recruitment was carried out from February to August 2011 and was facilitated via adverts in newspapers and flyers, radio, television, and social media. Inclusion criteria required participants who were healthy, non-smoking males and females aged 20 to 50 years who were currently engaged in at least part-time employment and/or undertaking a higher education or technical college course. They had no history of head injury or stroke, psychiatric or neurologic conditions, heart disease or diabetes and had no present kidney, liver or gastrointestinal conditions that might impair food metabolism. They were also free from any known or suspected food allergies. Individuals were ineligible to participate if they were pregnant or taking any form of herbal or vitamin supplement or over the counter or prescription medications (with the exception of the oral contraception pill).

### Treatments, randomisation and blinding

The treatment received (depending on gender) was either Swisse Men’s Ultivite F1®/Swisse Women’s Ultivite F1 ®(SMV) or matching placebo. The contents of the men’s and women’s SMV preparations are shown in Tables 1 and 2 respectively. Both contain a blend of vitamins at levels exceeding recommended daily intakes (RDI) [30] including B vitamins (e.g. Thiamin approx. 2500-4500% RDI, Riboflavin approx. 2300%-4545% RDI, Niacin approx. 185%-355% RDI, Pantothenic acid approx. 1070%-1700% adequate intake, B6 approx. 1900%-3165% RDI and B12 approx. 1250%-2080% RDI) and vitamins C (approx. 365% RDI), D (100% RDI) and E (approx. 330%-475% RDI), as well as minerals such as calcium, magnesium, potassium and iron. They also contain a range of antioxidants and extracts equivalent to approximately 2.6-3g of medicinal herbs including G*inkgo biloba*, V*itis vinifera*, S*ilybum marianum* and C*amellia sinensis*. Though the two formulations are predominantly equivalent the amount of some nutrients varies slightly, for example the women’s formula contains higher levels of calcium and iron, and there are a small number of herbal or plant extracts unique to either preparation. The placebo tablets were the same size and colour as the SMV tablets and contained starch and a small amount of riboflavin (2mg) designed to give a similar smell and colouration of the urine.

**Table 1:** Men’s SMV preparation contents

**Component**	**Daily dose**
Betacarotene	5mg
Vitamin D3	200IU
Vitamin E	50IU
Vitamin B1	30mg
Vitamin B2	30mg
Nicotinamide	30mg
Vitamin B5	64.13mg
Vitamin B6	24.68mg
Vitamin B12	30mg
Biotin	50 mcg
Folic acid	500mcg
Vitamin C	165.2 mg
Choline bitartrate	25mg
Inositol	25mg
Citrus bioflavonoid extract	40mg
Tyrosine	1 mg
Lysine	50mg
Calcium	21mg
Magnesium	57.89mg
Potassium	4mg
Iron	3mg
Chromium	6.2mcg
Manganese	1.2mg
Copper	28mcg
Iodine	50mcg
Zinc	6mg
Selenium	26mcg
Co-Enzyme Q10	1 mg
Spearmint oil	1.5mg
Parsley	10mg
Papaya	10mg
Lutein	200mcg
Celery	20mg
Astragalus	50mg
Buchu	10mg
Barberry	15mg
Gotu kola	50mg
Hawthorn	100mg
Horsetail	30mg
Fennel	15mg
Sarsparilla	50mg
Damiana	120mg
Ginger	5mg
Globe artichoke	50mg
Oats	500mg
Bilberry	25mg
Grape seed	1g
St Mary's thistle	50mg
Korean ginseng	50mg
Ginkgo	100mg
Saw Palmetto	200mg
Green tea	20mg
Tomato	700mg

**Table 2:** Women’s SMV preparation contents

**Component**	**Daily dose**
Betacarotene	5mg
Vitamin D3	200IU
Vitamin E	50IU
Vitamin B1	50 mg
Vitamin B2	50mg
Nicotinamide	50 mg
Vitamin B5	68.7 mg
Vitamin B6	41.14 mg
Vitamin B12	50 mcg
Biotin	50 mcg
Folic acid	500mcg
Vitamin C	165.2 mg
Choline bitartrate	25mg
Inositol	25mg
Citrus bioflavonoid extract	40mg
Lysine	50mg
Calcium	42 mg
Magnesium	47.16 mg
Potassium	2 mg
Iron	4.9 mg
Chromium	6.2mcg
Manganese	1.6 mg
Copper	58 mcg
Iodine	50.46 mcg
Zinc	5 mg
Selenium	26mcg
Co-Enzyme Q10	1 mg
Spearmint oil	1.5mg
Parsley	10mg
Papaya	10mg
Lutein	200mcg
Celery	20mg
Astragalus	50mg
Siberian Ginseng	25 mg
Bearberry	25mg
Gotu kola	10 mg
Hawthorn	30 mg
Horsetail	30mg
Fennel	15mg
Chamomile	15 mg
Licorice	10 mg
Ginger	15mg
Globe artichoke	50mg
Oats	500mg
Bilberry	25mg
Grape seed	1g
St Mary's thistle	50mg
Ginkgo	5mg
Green tea	20mg
Tomato	700mg

Treatment randomisation was independently carried out, separately for males and females, in blocks of four by the supplier; Swisse Vitamins Pty Ltd. Treatments were assigned a treatment identification number and packaged in identical boxes containing 18 blister packs of 7 tablets (18 x week supply). The extra 2-week supply of tablets was included to ensure continuous treatment in the event of late return visits and to aid in the assessment of treatment compliance. Following baseline assessment, randomised participants were provided with the treatment and instructed to take one tablet daily with, or immediately following breakfast for the next 16 weeks, and to bring all unused tablets to each study visit. Data were unblinded only after all data were finalised and preliminary analyses were complete.

### Procedure

Participants attended a brief practice session during which written consent was obtained, eligibility was confirmed and they were familiarised with all study measures. Demographic characteristics that may have modified outcomes were recorded, including age, education and BMI and measures of trait anxiety (State-Trait Anxiety Inventory) [31], depression (Beck Depression Inventory – II) [32] and intelligence (WASI Matrix reasoning task) [33] were completed. Three testing sessions were undertaken: at baseline, Week 8 and Week 16. As this trial was concerned with chronic effects of supplementation, participants were asked to abstain from taking their tablet on the morning of testing sessions. This avoided any potentially confounding acute effects that a single day’s treatment may have had on assessments and which could not have been differentiated from the chronic effects of repeated and prolonged supplementation.

Participants completed standardised assessments of wellbeing, mood and stress, and a computerised cognitive test battery. Blood measures, cardiovascular function and salivary cortisol levels were investigated as potential mechanisms of action. In total, each testing session lasted approximately 3 hours. A researcher from the Centre of Human Psychopharmacology at Swinburne University of Technology administered all tests. The number of tablets returned was used to calculate total treatment compliance, with participants being required to be at least 80% compliant for inclusion of their data in analyses.

### Qualitative assessment component

In addition to mood, cognitive and biomarker assessments, at the conclusion of the final testing session (week 16), participants were presented with three written, semi-structured, open-ended qualitative questions. Pre-study, a pilot form was constructed via a consensus between researchers, being tested on a sample of colleagues for feedback on the utility of its format. In response to the feedback the form was slightly modified by consolidating questions into three major areas of enquiry. For each question, participants were directed to indicate whether or not they perceived any direct effects from taking the tablets, and if so to describe their experience in as much detail as possible.

The questions were as follows:

1) “*In the past month describe any positive effects (if any) on your physical or mental health (mood, stress, brain function) that you think may have occurred due to taking the tablets.”*

2) “*In the past month describe any negative effects (if any) on your physical or mental health (mood, stress, brain function) that you think may have occurred due to taking the tablets.”*

3) “*If apparent, please describe any unusual effects that you think may have occurred from taking the tablets.”*

### Data analysis

As this was a double-blind study neither the study researchers or the participants were aware of what treatment the participant had been receiving. After the study was completed and the data were available for analysis, the question transcripts were read by two researchers and coded according to a grounded theory approach to data analysis. Initially, participants’ experiences were separated into “positive” and “negative” effects. Following this, two major domains (mental/cognitive and physical) were categorised, within which many distinct themes were identified via a thematic analytic approach. Each reported experience was then coded under a theme heading by the researchers in a blinded manner. The themes were then submitted to researcher triangulation (whereby the three researchers provided independent readings of the written responses, with any divergences of interpretation discussed until resolution is reached).

To provide increased scientific rigour, a quantitative analysis was performed to compare the frequency with which themes were reported within treatment groups. For each participant every theme was coded as having been “reported” or “not reported”. Using this categorical classification a Chi Square analysis was then conducted for each theme, with significance set at a *p* <.05, while a trend towards significance regarded as between *p* of .05 and .10. These analyses were then repeated separately within genders.

## Results

### Sample

**Figure 1:**
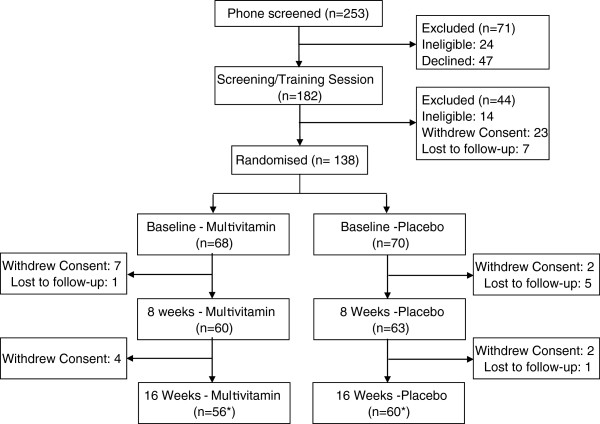
**Trial design and visit completion.** *One participant from the Multivitamin group and one participant from the Placebo group failed to provide qualitative data.

A total of 182 participants were enrolled in the study, of these 138 received treatment. An attrition rate of approximately 16% was observed, with 116 participants completing the study (Figure 1). Qualitative data was available from 114 participants, 59 from the placebo group and 55 from the SMV group. A count of returned tablets indicated that all participants satisfied the 80% compliance requirement at their final visit. The characteristics of the sample (Table 3) revealed that the mean age of participants was early 30s, they were educated and had higher than average intelligence, low depression and anxiety levels, and an average BMI (for adult Australians). The two treatment groups did not differ significantly on any examined demographic characteristic, either in the sample as a whole or when split by gender.

**Table 3:** Baseline characteristics of participants (n=114)*

**Gender**	**Age**	**Education**	**Intelligence**	**BMI**	**Trait Anxiety**	**Depression**
**Placebo**						
Male	30.93 (7.47)	17.00 (2.57)	28.43 (2.70)	26.13 (4.98)	31.00 (6.15)	3.82 (3.78)
Female	30.80 (6.46)	17.71 (3.14)	28.87 (2.25)	22.03 (5.59)	34.52 (7.84)	3.71 (4.04)
Total	30.86 (6.90)	17.37 (2.88)	28.66 (2.46)	23.98 (5.65)	32.85 (7.25)	3.76 (3.89)
**Multivitamin**						
Male	28.91 (6.90)	16.38 (1.86)	29.29 (3.18)	24.41 (3.21)	34.54 (9.43)	5.29 (4.67)
Female	32.94 (7.85)	17.71 (3.07)	28.13 (2.91)	23.98 (5.31)	32.68 (7.13)	4.61 (5.04)
Total	31.18 (7.76)	17.13 (2.67)	28.65 (3.06)	24.47 (4.46)	33.49 (8.18)	4.91 (4.85)
**All**						
Male	30.00 (7.20)	16.71 (2.27)	28.83 (2.94)	25.34 (4.30)	32.63 (7.96)	4.50 (4.24)
Female	31.87 (7.21)	17.71 (3.08)	28.51 (2.60)	22.99 (5.50)	33.60 (7.49)	4.16 (4.56)
Total	31.02 (7.24)	17.25 (2.77)	28.65 (2.75)	24.07 (5.10)	33.16 (7.69)	4.32 (4.40)

* Data from participants randomized conditions; Data depicted as means and standard deviations; Education= Years of study; Intelligence = Matrix reasoning score; Trait Anxiety = State-Trait Anxiety Inventory–Trait Scale Score; Depression = Beck Depression Inventory-II score.

### Overview of qualitative data

The qualitative data were divided into “Positive Effects” or “Negative Effects” and coded into four primary domains, in addition to supplementary analysis of any unusual experiences. A total of 21 further emergent themes were found and coded under these main domains (see Table 4). Examples of participants’ experiences are detailed below. Statements are provided that best represent the themes revealed from qualitative analysis. As a result, some participants’ responses are not included, while others are quoted more than once.

**Table 4:** Experiential themes identified

**Positive experience**	**Negative effect**
**Mental/Cognitive Domain**	**Mental/Cognitive Domain**
General Mental Benefit	Impaired Concentration/Attention
Improved Concentration, Attention &/or Memory	Stressed or Anxious
Increased Calmness or Relaxation	More Moody and Emotional
Better Mood & Emotional State	**Physical Domain**
**Physical Domain**	Impaired Sleep
General Physical Benefit	More Fatigued &/or Drowsy
Improved sleep	Poorer Health &/or Immunity
More Energetic &/or Alert	Upper Gastrointestinal complaint
Better Health &/or Immunity	Indigestion or Nausea
Dermatological Benefit	Dermatological Complaint
Increased/Better Appetite	Gynaecological Effect
	Weight Gain

### Summary of positive and negative experiences

In response to the question posed to participants about any positive effects, the majority of participants (60.0%) in the SMV group reported at least one positive experience compared to those in the placebo group (50.8%; Table 5). In the area of positive reported effects, the two main statistically significant themes identified in the SMV group over the placebo group were an increase in energy and mental alertness, and an increase in mood and mental/emotional wellbeing. More people in the SMV group reported sleep improvement compared to placebo, but due to low power the result did not reach statistical significance. Curiously, two participants reported a beneficial change in appetite, while one person (placebo group) said their skin was better. Of the negative effects reported, no significant differences were found between SMV and placebo (further detail below).

**Table 5:** Differential positive and negative effects experienced by participants

	**Full sample**		**Males**		**Females**	
	**Placebo**	**Multi**	**Placebo**	**Multi**	**Placebo**	**Multi**
	**n = 59**	**n = 55**	**n = 28**	**n = 24**	**n =31**	**n = 31**
**Positive experience**	**50.8%**	**60.0%**	**64.3%**	**62.5%**	**38.7%**	**58.1%**
**Mental/Cognitive Domain**						
General Mental Benefit	8.5%	9.1%	7.1%	0.0%	9.7%	16.1%
Improved Concentration, Attention &/or Memory	8.5%	9.1%	10.7%	20.8%	6.5%	0.0%
Increased Calmness or Relaxation	20.3%	14.5%	21.4%	25.0%	19.4%	6.5%
Better Mood & Emotional State	8.5%	23.6% *	7.1%	16.7%	9.7%	29.0% **#**
**Physical Domain**						
General Physical Benefit	8.5%	5.5%	14.3%	12.5%	3.2%	0.0%
Improved Sleep	5.1%	15.5% **#**	3.6%	8.3%	6.5%	19.4%
More Energetic &/or Alert	11.9%	29.1% **	17.9%	29.2%	6.5%	29.0% ***
Better Health &/or Immunity	6.8%	10.9%	10.7%	8.3%	3.2%	12.9%
Dermatological Benefit	1.7%	0.0%	3.6%	0.0%	0.0%	0.0%
Increased/Better Appetite	1.7%	1.8%	3.6%	4.2%	0.0%	0.0%
**Negative experience**	**28.8%**	**30.9%**	**25.0%**	**25.0%**	**32.3%**	**35.5%**
**Mental/Cognitive Domain**						
Impaired Concentration/Attention	5.1%	3.6%	3.6%	4.2%	6.5%	3.2%
Stressed or Anxious	1.7%	3.6%	0.0%	0.0%	3.2%	6.5%
More Moody and Emotional	1.7%	5.5%	3.6%	0.0%	0.0%	9.7%
**Physical Domain**						
Impaired Sleep	1.7%	1.8%	3.6%	0.0%	0.0%	3.2%
More Fatigued &/or Drowsy	6.8%	5.5%	7.1%	8.3%	6.5%	3.2%
Poorer Health &/or Immunity	1.7%	1.8%	3.6%	0.0%	0.0%	3.2%
Upper Gastrointestinal complaint	1.7%	9.1%	0.0%	8.3%	3.2%	9.7%
Indigestion or Nausea	1.7%	3.6%	0.0%	4.2%	3.2%	3.2%
Dermatological Complaint	1.7%	1.8%	0.0%	4.2%	3.2%	0.0%
Gynaecological Effect	0.0%	3.6%	-	-	0.0%	6.5%
Weight Gain	3.4%	1.8%	3.6%	0.0%	3.2%	3.2%

*χ^2^_(1)_= 4.921, *p* = 0.027; ** χ^2^_(1)_= 5.245, *p* = 0.022; *** χ^2^_(1)_ = 5.415, *p* = 0.020 ^#^*p* = 0.05-0.10 (trend).

### Multivitamin effects on energy levels

A total of 17.2% more participants in the SMV group significantly reported increased energy levels and mental alertness compared to the placebo group (*p* =.022). Females in the SMV group significantly (*p* =.020) reported experiences of increased energy and mental alertness than females in the placebo group (22.5% difference). Positive perceived effects on participant’s energy levels commonly reported in the SMV group included:

"“More alert, bright, full of energy”"

"“I found getting up early…easier”"

"“Have felt like I have more energy”"

"“I feel some sort of relaxation and energy”"

"“My energy levels were quite good over the period and I felt happy most of the time”"

"“A general feeling of more alertness particularly in the morning”"

"“I felt physically less tired”"

### Multivitamin effects on mood and stress

Compared with the placebo group, a total of 15.1% more participants in the SMV group reported the experience of better mood and emotional state. This difference was statistically significant (*p* = .027). While nearly 20% more female participants reported an improvement of mood in the SMV compared to control, the result did not reach significance (*p* =.054). Examples of beneficial experiences reported by the SMV group in the area of enhanced mood and reduced stress were:

"“I feel a bit more carefree and I stress less”"

"“I have felt more relaxed; mood is more stable, less ups and downs”"

"“Stress levels have reduced… Feel calmer”"

"“Mood more uplifted”"

"“Much more calm than before taking this”"

"“Mood-wise I think I am more patient”"

### Multivitamin effects on sleep

Another theme that emerged was the experience by some participants of an improvement in their sleep. While 10% more participants in the SMV group reported a positive effect on sleep, this was just outside of significance (*p* =.087). Examples of beneficial effects in the SMV group:

"“Less tired/easier to fall asleep""

"“Sleep quite peaceful… Increased my sleep during the night”"

"“Felt that tablets assisted in helping to sleep”"

"“Better sleep, less stress, more energy during the day time”"

### Perceived negative experiences

Importantly, no major adverse effects or reactions emerged from our thematic analysis, providing additional support for the quantitative finding that SMV was well tolerated by this sample. Overall SMV was well-tolerated with no statistical difference in the number of overall negative effects reported between the placebo (28.8%) and MV (30.9%) groups. Negative mental effects were not different between groups. Specifically, three females reported feeling more ‘moody’ or ‘emotional’ compared to none in the placebo group. Five participants experienced impaired concentration/alertness (two SMV, three placebo) three were more stressed or anxious (two SMV, one placebo), however this was not significantly different between groups.

Several participants reported experiencing mild negative physical reactions that occurred during the study. The most common complaint related to participants feeling dehydrated or need to drink soon after taking the tablets; experiencing heartburn; or an unpleasant taste. While 7% more participants in the SMV than the placebo group reported mild negative gastrointestinal effects, this was just outside of statistical significance (*p*=.077). Another negative experience included two females in the SMV noting they had a negative gynaecological effect (one perceived an increase in premenstrual symptoms, and the other a change in her menstrual cycle). Two participants in the placebo group and one participant in the multivitamin group reported weight gain. Impaired sleep, dermatological complaints (rash or dry skin/nails) and more frequent illness were each reported by single participants in both groups.

### Unusual experiences reported

Six unusual effects were reported by participants, although it cannot be determined whether these experiences were related to either group’s intervention. For the SMV group, three unusual effects were reported: *“Early on I felt that I sweated more - under the arms” “Faeces more hard perhaps” “Sensitive to the room temperature when sleeping*”.

A total of 3 people in the placebo group reported unusual effects: *“I experienced fuzzy eyes” “Several mornings I woke up with a headache”* “*Kidney-ache occurred for the last six weeks… Quite intense about 4 weeks ago”*.

## Discussion

Results of the first exploratory qualitative assessment of participants’ experiences of chronic daily administration of a multivitamin (MV) revealed significant effects in several areas. The major significant themes found between SMV and placebo concerned participants’ perceived increased energy levels and enhanced mood, with a trend towards some experiencing better sleep. We performed a double-blind analysis and utilised quantitative methods of data analysis (i.e. Chi square test), increasing confidence in these results.

While assessment on quantitative numerical assessment scales is considered the “gold standard”, use of qualitative methods, involving data from open-ended questions, clinician’s notes, and forums, may reveal outcomes that may not have been found by established quantitative assessment tools. This method has been used successfully in other nutraceuticals studies [34,35], but not yet for MV studies. As commented by Berk et al. [35], unexpected but important phenomena may escape detection in purely quantitative studies. In their double-blind RCT involving the application of N-acetyl cysteine for schizophrenia, emergent themes arose which had not been captured by the quantitative rating scales utilised. Such identified novel effects can potentially factor into future practice guidelines, or can be specifically studied in subsequent RCTs.

The finding of participants reporting significantly increased mood and energy is in line with results of previous MV and B vitamin complex research; adequate B vitamin levels are critical for neuronal communication and energy generation. Importantly, very few participants experienced any negative effects, and no significant adverse reactions were identified in the study. The only cases of identified side effects concerned minor gastrointestinal symptoms; reactions such as nausea have been previously reported by a small percentage of the general public who take a MV [36]. Analysis by gender revealed that the observed benefit on mood and energy were more likely to be experienced by women than men.

Limitations of the qualitative component are acknowledged. The use of a written semi-structured assessment form does not provide the rich exploration of experience that can achieved from interviews with open-ended questions or focus groups, and the small sample size meant that only very common effects of SMV could be reliably detected. Additionally as the qualitative questions addressed perception of change due to treatment they were only completed at the final testing visit. An intent-to-treat analysis was therefore not possible for this data and only study completers were included in the analysis.

It should be noted that these analyses have not be corrected for multiple comparisons. When multiple outcome measures, such as our 21 themes, are assessed, a p-value correction may not always be the best approach as it can result in an overly conservative significance level and increased likelihood of a Type 2 error [37]. The themes seen here to benefit from multivitamin use are supported theoretically and by the supplementation effects seen in previous quantitative studies.

The qualitative data presented here provides the first exploration of the important topic of participants’ experiences and should prompt further, more in-depth investigation of this topic as part of future RCTs.

## Conclusions

This paper represents the first documented qualitative investigation of participants’ experience of chronic administration of a multivitamin. Overall the exploratory experiential data provided by the participants was found to reflect the general findings of previous quantitative trial data; multivitamin supplementation may be associated with appreciable mood enhancement and increases in energy even in a normal, non-depressed and non-anxious population. Future RCTs are encouraged to adopt a similar mixed-methods approach.

## Abbreviations

MV: Multivitamin; RCT: Randomised controlled trial; SMV: Swisse Men’s Ultivite F1®/Swisse Women’s Ultivite F1 ®.

## Competing interests

The National Institute of Integrative Medicine, of which Professor Avni Sali is currently director, receives financial support from Swisse Vitamins Pty Ltd. Andrew Pipingas and Avni Sali are currently members of the Scientific Advisory Board for Swisse Vitamins Pty Ltd. Aside from oversight of study design and provision of supplements, Swisse Vitamins Pty Ltd were not involved in any other aspects of the conduct of the trial including analysis, or interpretation of the trial findings.

## Authors' contributions

JS contributed to data analysis and interpretation and prepared the manuscript. KC collected data, conducted data analysis and interpretation and contributed to the manuscript writing. DAC, AS, CS, MK, AS and AP contributed to study conception and design and to manuscript revision. DJW and EF collected data and contributed to manuscript revisions. All authors have read and approved the final version of the manuscript.
